# Supplementary material for: Comparison of Cultures and 16S/18S Amplicon-Based Microbiome Analyses for Diagnosing Nosocomial Pneumonia in Patients Admitted to the Intensive Care Unit—An Exploratory Study
Source: Diagnostics (Basel). 2025 Dec 15;15(24):3202. doi: 10.3390/diagnostics15243202 (PMC12731414; doi:10.3390/diagnostics15243202)
Supplement: Supplementary file 1 [file diagnostics-15-03202-s001.zip › Supplementary Table S1.pdf]

Supplementary Table S1. Antibiotic treatment before ICU admission, before the BAL was performed and after the BAL was performed

Patient number	AB_preICU	AB_preBAL	AB_postBAL
1	piperacillin/tazobactam+metronidazol	piperacillin/tazobactam+metronidazol	piperacillin/tazobactam+metronidazol +fluconazol
2	cefuroxim+metronidazol+clarithromycin+fluconazol	meropenem+fluconazol	meropenem+vancomycin+fluconazol
3	piperacillin/tazobactam+fluconazol +vancomycin	meropenem+ciprofloxacin+fluconazol+vancomycin	meropenem+ciprofloxacin+fluconazol +vancomycin
4	meropenem+metronidazol+fluconazol	meropenem+metronidazol+fluconazol	meropenem+metronidazol+anidulafungin
5	cefuroxim+ciprofloxacin	cefuroxim+ciprofloxacin	meropenem+ciprofloxacin+caspofungin
6	piperacillin/tazobactam	piperacillin/tazobactam	piperacillin/tazobactam+ampicillin
7	dicloxacillin	piperacillin/tazobactam+metronidazol+dicloxicillin	piperacillin/tazobactam+metronidazol +ciprofloxacin
8	piperacillin/tazobactam+metronidazol	piperacillin/tazobactam+metronidazol	piperacillin/tazobactam+metronidazol
9	piperacillin/tazobactam+ciprofloxacin	piperacillin/tazobactam+ciprofloxacin	piperacillin/tazobactam+dicloxacillin+ caspofungin
10	piperacillin/tazobactam+ciprofloxacin+metronidazol	piperacillin/tazobactam+ciprofloxacin+metronidazol	piperacillin/tazobactam+metronidazol
11	piperacillin/tazobactam+metronidazol	meropenem+metronidazol+ciprofloxacin+clarithromycin	meropenem+metronidazol+ciprofloxacin+clarithromycin
12	piperacillin/tazobactam	piperacillin/tazobactam	piperacillin/tazobactam+vancomycin

13	penicillin+clindamycin	piperacillin/tazobactam+clarithromycin+clindamycin+metronidazol	meropenem
14	piperacillin/tazobactam	piperacillin/tazobactam	piperacillin/tazobactam
15	meropenem+fluconazol	meropenem+vancomycin+fluconazol	meropenem+vancomycin+anidulafungin
16	piperacillin/tazobactam	piperacillin/tazobactam	piperacillin/tazobactam+clarithromycin.
17	piperacillin/tazobactam	piperacillin/tazobactam	piperacillin/tazobactam+clarithromycin
18	piperacillin/tazobactam	piperacillin/tazobactam+clarithromycin	meropenem+fluconazol
19	piperacillin/tazobactam+clarithromycin	piperacillin/tazobactam+clarithromycin	piperacillin/tazobactam
20	piperacillin/tazobactam	piperacillin/tazobactam	piperacillin/tazobactam
21	piperacillin/tazobactam+clarithromycin	piperacillin/tazobactam+clarithromycin	piperacillin/tazobactam+clarithromycin
22	piperacillin/tazobactam+clarithromycin	meropenem	meropenem
23	None	piperacillin/tazobactam + clarithromycin	piperacillin/tazobactam+clarithromycin

Abbreviations: AB = antibiotics, preICU = before admission at intensive care unit, preBAL= before the BAL was performed, postBAL= after the BAL was performed
