# Supplementary material for: Comparison of Cultures and 16S/18S Amplicon-Based Microbiome Analyses for Diagnosing Nosocomial Pneumonia in Patients Admitted to the Intensive Care Unit—An Exploratory Study
Source: Diagnostics (Basel). 2025 Dec 15;15(24):3202. doi: 10.3390/diagnostics15243202 (PMC12731414; doi:10.3390/diagnostics15243202)
Supplement: Supplementary file 1 [file diagnostics-15-03202-s001.zip › Supplementary Table S2.pdf]

Table S2 The microbiological findings in BAL and TS using both culture and Microbiome analyses

Patient number	Culture		Microbiome	
	**BAL**	**Tracheal suction**	**BAL**	**Tracheal suction**
1	*C. albicans* +	*C. albicans* +	*C. albicans*	*C. glabrata*
2	Negative	*C. albicans* +, *C. tropicalis* + *E. faecalis* +, *S. haemolyticus*	No DNA	*S. epidermidis*
3	*C. albicans* +,	*C. albicans* +, *S. epidermidis* +	Negative	*C. albicans*
4	*C. glabrata* +, *S. haemolyticus* +	*C. glabrata* +, *S. haemolyticus* +	*C. glabrata*	*S. haemolyticus*
5	*S. epidermidis* +, *C. dubliniensis* +	*S. epidermidis* +, *C. dubliniensis* +	No DNA	No DNA
6	*K. pneumoniae* ++, *E. coli* ++	*E. coli* ++, *K. pneumoniae* +++	*E. coli*	*E. coli*
7	*C. albicans* ++, *C. glabrata* +, *K. pneumoniae*+	*C. albicans* ++, *C. glabrata* +, *K. pneumoniae*+	*C. glabrata*	*C. glabrata*
8	*K. pneumoniae* +++, *E. faecium* +++, *C. glabrata* +++, *C. albicans* +	*K. pneumoniae* +++, *E. faecium* +++, *C. glabrata* +++, *C. albicans* +	*K. pneumoniae*	*K. pneumoniae*
9	*C. tropicalis* ++, *C. albicans* ++, *S. aureus* +	*C. tropicalis* ++, *C. albicans* ++, *S. aureus* ++	*P. canis*	*P. canis*

	E cloacae +,<br>*E. coli* +,			
10	Negative	*C. albicans* ++	Negative	*C. albicans*
11	*E. coli* +++,<br>*K. pneumoniae* +	*E. coli* +++,<br>*K. pneumoniae* +	*E. coli*	*E. coli*
12	*S. epidermidis* +	*S. epidermidis* +,<br>*S. haemolyticus* +	Negative	Negative
13	*C. parapsilosis* +	*C. parapsilosis* +,<br>*C. krusei* +	Negative	Negative
14	*Neisseria* spp. +,<br>*S. epidermidis* +	*C. albicans* +	*S. pseudopneumoniae*	Negative
15	*S. epidermidis* +	*S. hominis* +	Negative	Negative
16	*C. tropicalis* +	*C. tropicalis* ++,<br>*S. epidermidis* +	Negative	Negative
17	*C. albicans* +	*C. albicans* +	Negative	Negative
18	Negative	Negative	*Ps. flourescens*<br>*S. marscescens*	*H. influenzae*
19	Negative	Negative	Negative	Negative
20	*S. epidermidis* +	Negative	*T. whipplei*	*T. whipplei*
21	*E. coli* +,<br>*K. variicola* +,<br>*C. spp.* +	*E. coli* +,<br>*K. variicola* +,<br>*C. spp.* +	*E. coli*	*E. coli*

22	*E. faecalis* +,<br>*S. lugdunensis* +,<br>*S. epidermidis* +,	*E. faecalis* +,<br>*S. lugdunensis* +,<br>*S. anginosus* +,	*S. epidermidis*	*S. epidermidis*
23	Negative	Negative	Negative	Negative

Abbreviations: S. epidermidis – *Staphylococcus epidermidis; S. haemolyticus – Staphylococcus haemolyticus; S. lugdunensis – Staphylococcus lugdunensis; S. aureus – Staphylococcus aureus; S. pseudopneumoniae – Streptococcus pseudopneumoniae; S. anginosus – Streptococcus anginosus; E. faecalis – Enterococcus faecalis; E. faecium – Enterococcus faecium; E. coli – Escherichia coli; K. pneumoniae – Klebsiella pneumoniae; K. variicola – Kelbsiella variicola; E. cloacae – Enterobacter cloacae; P. canis – Pasteurella canis; Ps. fluorescens – Psedomonas fluorescens; S. marscescens – Serratia marscescens; T. whipplei – Tropheryma whipplei; C. albicans – Candida albicans; C. glabrata – Candida glabrata; C. tropicalis – Candida tropicalis; C. dubliniensis – Candida dubliniensis; C. parapsilosis – Candida parapsilosis; C. spp. – Candida spp.*

The growth rate is indicated as: + weak growth, ++moderate growth, +++strong growth
